# Advances in colon capsule endoscopy: a review of current applications and challenges

**DOI:** 10.3389/fgstr.2023.1316334

**Published:** 2023-11-17

**Authors:** E. Gibbons, O. B. Kelly, B. Hall

**Affiliations:** Gastroenterology Department, Connolly Hospital, Dublin, Ireland

**Keywords:** colon capsule endoscopy, colorectal cancer, colorectal cancer surveillance, noninvasive surveillance, bowel screen

## Abstract

Colon capsule endoscopy (CCE) has been demonstrated to be comparable to traditional colonoscopy and better than CT colonography (CTC) for the detection of colonic pathology. It has been shown to have a high incremental yield after incomplete colonoscopy. It is a safe test with good patient acceptability. Challenges currently include great variability in completion rates and high rates of re-investigation. In this review, we will discuss the evidence to date regarding CCE in symptomatic and surveillance populations, and in those post incomplete colonoscopy. We will discuss current challenges faced by CCE and areas for further research.

## Introduction

The development of capsule endoscopy has modified our approach to the diagnosis of GI disease. Since its inception in the late 1990’s, the relatively rapid uptake of small bowel capsule endoscopy (SBCE) as an important clinical tool can be largely ascribed to a number of key factors. It was designed as a non-invasive, ambulatory method of viewing the entire small bowel without the need for sedation, a day-ward bed or invasive endoscopy. There is no radiation associated risk. It is a relatively easy examination to perform in an outpatient setting and is superior when compared with endoscopic or radiological procedures in numerous situations, most notably obscure gastrointestinal bleeding (1–3).

CCE is a relatively newer technique. It is a non-invasive and safe method of viewing the large bowel, and represents an alternative to traditional colonoscopy and CT colonography (CTC). The PillCam system by Medtronic is the most widely referenced colon capsule to date. Results from their first generation colon capsules were disappointing with regard to polyp detection and completion rates when compared to colonoscopy or CTC (4–10). These studies prompted the development of a second-generation system with increased capsule frame rate and improved field of vision. A meta-analysis by Spada et al. comparing the first- and second-generation capsules in 2016 found both sensitivity and specificity increased substantially with the second generation (11). Other manufacturers with commercially available colon capsules include Jinshan’s OMOM system and Stratis Medical’s MiroCam in the United States. There are no studies comparing the efficacy of these various systems.

Guidance from the European Society of Gastrointestinal Endoscopy (ESGE), updated in 2020, provided the first framework for healthcare providers for indications, preparation for, and reporting of CCE (12). This consensus document advises that CCE is safe, appears accurate in average-risk individuals, and is appropriate for use in those with incomplete colonoscopy and without stenosis. CCE use is recommended if colonoscopy is not deemed appropriate or not possible. It is advised that there remains a paucity of studies based in the setting of screening, and in comparing CCE with radiological imaging or traditional endoscopic modalities (12).

## Polyp detection and colorectal cancer surveillance

The adenoma-carcinoma sequence describes the transformation of normal colorectal epithelium to adenomatous polyp to invasive cancer. This temporal sequence of events offers an opportunity for endoscopic intervention at a precancerous stage. A well designed screening programme can detect disease early, allowing timely intervention by polypectomy at endoscopy (13). Despite this, CRC is currently the third most common cancer, and the second leading cause of cancer related deaths in the world (14). It is the third most common cause of cancer in Ireland, and accounts for 11% of all cancer related deaths (15).

Fecal immunohistochemical testing and traditional colonoscopy are the most commonly used screening tools worldwide currently, with CTC being used in certain subgroups (14). Colonoscopy remains the gold standard, enabling both diagnostic and therapeutic interventions. National screening programmes, such as the National Colorectal Cancer Screening Service (Bowel Screen) in Ireland, face significant challenges in attempting to deliver timely endoscopy. This has been exacerbated in recent years by the COVID-19 pandemic, but demographic changes alone are expected to lead to an increase in new CRC cases by 79% between 2012 and 2035 worldwide (16, 17). Recent change in guidance in the United States to begin screening from the age of 45 years will add an estimated 20 million individuals in the 45- 49 year age group to the screening population (18).

To date, CCE is playing a minor role in screening but there is a growing body of evidence supporting its use (19–21). Both the UK and Denmark have, in recent years, included CCE in their national CRC surveillance pathways and a wealth of data regarding its efficacy is expected in the coming years from these pilots (22). A recent systematic review of CCE use in a screening population by Vuik et al. identified 582 studies published up to September 2020, of which 13 were included, comprising 2485 patients (20). Eight studies used CCE as a filter test after a positive FIT result and five studies used CCE for primary screening. The polyp detection rate of CCE was 24% - 74%. For polyps > 6 mm, sensitivity of CCE was 79% - 96% and specificity was 66% - 97%. For polyps ≥ 10 mm, sensitivity of CCE was 84% - 97%, which was superior to CTC. The CRC detection rate for completed CCEs was 93%. The authors concluded that the accuracy of CCE was comparable to colonoscopy and superior to CTC, making it a good alternative modality for screening programmes. A more recent meta-analysis by Kjolhede et al. comparing polyp detection rates between CCE and colonoscopy concluded that CCE demonstrated high sensitivity and specificity for per-patients polyps compared to colonoscopy (19).

Back-to-back comparison of CCE and colonoscopy in surveillance populations has been performed in few small studies (23–27). Kobaek et al. compared the two modalities and found CCE, when complete, to have a superior polyp detection rate (PDR) of 86% versus 65% at colonoscopy (p < 0.001) (24). In another cohort, Holleran et al. showed good correlation between CCE and colonoscopy for any lesion with Cohen’s kappa of 0.62, and in their cohort use of CCE would have potentially reduced the number of colonoscopies required by 71% (23). Spada et al. analyzed 109 participants who underwent back to back procedures. CCE sensitivity for polyps of at least 6 mm was 84% and 88% for polyps at least 10mm in size, with a specificity of 64% and 95% respectively (25). In a pilot study by Spinzi et al, subjects participating in a national CRC surveillance programme underwent CCE, followed by both colonoscopy and CTC at day 15 (27). The primary end point was to evaluate CCE and CTC accuracy for identification of polyps > 6mm. Both CTC and CCE performed well with sensitivities of 88.2% and 88.2%, respectively, and specificities of 84.8% and 87.7% respectively. The main difference this study found was in patient preference and acceptability of the test with 78% selecting CCE as their preferred procedure.

In a recent systematic review and meta-analysis, miss rates of 9% for advanced adenomas, 27% for serrated polyps and 34% for flat adenomas at colonoscopy were calculated (28). Data regarding CCE miss rates and detection of more challenging polyps remains limited. Two studies have looked at the ability of CCE to detect laterally spreading tumors (LSTs) (29, 30). LSTs are non-polypoid lesions > 10mm which extend laterally and circumferentially along the colonic wall rather than vertically (31). These lesions are typically more challenging to detect at traditional colonoscopy (32). They reported lower sensitivity of CCE for LSTs than colonoscopy (29, 30). CCE appears to have a higher accuracy than colonoscopy for cancer, however (11, 23, 25, 33, 34). To date only one case report has been published documenting a missed cancer at complete CCE (35).

## Management post incomplete colonoscopy: current practices and role of CCE

Incomplete colonoscopies can occur in up 20% of patients and are associated with higher rates of missed lesions (36, 37). Completion depends on both the technical expertise of the endoscopist and patient tolerability. Tortuous, redundant colons or patients with a history of abdomino-pelvic surgery often present a challenge to even the experienced endoscopist (37–39). CTC is typically considered first line investigation after an incomplete colonoscopy, with numerous studies reporting CTC sensitivity to be comparable to colonoscopy (40–42). Repeat colonoscopy with extended bowel preparation, or referral to a more experienced endoscopist are alternative approaches (43). ESGE and ASG guidance now endorse the use of CCE in this situation (12, 43).

Four prospective studies have looked specifically at this group, with large numbers of polyps identified by CCE in areas not reached by initial colonoscopy (44–47). The study by Nogales et al. comprised 96 patients who had an incomplete colonoscopy and went on to have a CCE for completion (45). CCE revealed new lesions in 58 patients (60.4%) at locations not previously reached by colonoscopy. Balte’s prospective multicenter study included 74 patients who underwent CCE after colonoscopy, either the following day following extended prep or at a later date (44). CCE visualized mucosa missed by colonoscopy in 90% of those who underwent CCE the following day, and in 97% of those who underwent delayed CCE (44). In the third, a per-patient analysis for polyps > 6mm, CCE detected polyps in 24 patients (24.5%) and CTC in 12 patients (12.2%) (46). Hussey et al. looked at same day CCE after incomplete colonoscopy in fifty patients. CCE had a significant diagnostic yield of 74%, with an incremental yield of 38% (47).

CCE performs well compared to CTC with regards to polyp detection (48–50). A meta-analysis by Deding et al. comparing CCCE and CTC in those with incomplete colonoscopy found the completion rate was lower in CCE than CTC, but CCE had a higher diagnostic yield, almost fourfold, for polyps of any size (48). A prospective, single-center, randomized trial, the VICOCA study compared CCE and CTC in 290 individuals, using colonoscopy as the gold standard (49). It reported sensitivity, specificity and positive and negative predictive values of CCE for the detection of patients with any neoplastic lesion of 98.1%, 76.6%, 93.7% and 92.0%, respectively. CTC had sensitivity, specificity and positive and negative predictive values of 64.9%, 95.7%, 96.8% and 57.7%, respectively. In terms of detection of polyps > 6mm, the sensitivity of CCE and CTC was 96.1% and 79.3%. CCE was shown have superior sensitivity for detecting serrated lesions (73.6% versus 32.9%; p < 0.001) (49). Similarly the TOPAZ trial concluded that CCE should be considered comparable, if not superior, to CTC as a screening test (50).

## Bowel preparation for CCE

A good bowel preparation protocol will achieve adequate colonic cleanliness, reduce effects of colonic bubbles and aid timely capsule excretion (51). The quality of the preparation protocol is dependent on many factors, including, laxative used, timing and volume of laxative, types of boosters used, timing of boosters, use of prokinetics and patient tolerability. Great variability exists between studies with regard to bowel cleanliness and excretion rates (20, 51). A recent meta-analysis of the diagnostic accuracy of CCE versus traditional colonoscopy for polyp detection found capsule excretion rates ranging from 57%- 100% and adequate bowel prep ranging from 40%-100% concluding that improvements in both adequate cleanliness rates and excretion rates are required before widespread implementation of CCE into CRC surveillance programmes (19). A systematic review by Bjoersum-Meyer found completion rates, meaning achievement of adequate colonic cleanliness with excretion of capsule prior to battery dying, to be suboptimal in almost all studies included (51).

Early protocols combined high volume (4 liter) polyethylene glycol (PEG) cleansing solutions and sodium phosphate (NaP) boosters (5–8, 33, 52, 53). NaP boosters achieve high excretion rates but concerns regarding nephrotoxicity, even in those without a history of renal insufficiency, have been documented (7, 8, 23, 54–58). Though the ESGE still advocates for NaP-based boosters for CCE in most recent guidance, the trend is towards avoidance of these boosters, with a 2021 meta-analysis reporting only 24% studies using NaP in the four years preceding review, versus >60% in the years prior to this (51). More recently, a combination of lower volume PEG preparations, NaP free boosters and prokinetics are being utilized with good success, reducing the volume of fluid patients have to ingest without compromising polyp detection (26, 58). Prucalopride, a serotonin receptor antagonist that accelerates colonic transit time, and castor oil, a vegetable oil from the castor bean, have recently been shown to significantly improve CCE completion and polyp detection rates (59–63). There is widespread variation in preparation protocols used in clinical practice and a need for large, prospective studies comparing these protocols (51).

A validated scoring system for reporting of bowel cleanliness is not routinely used to date and great inter-observer variability exists (64). CC-Clear is a novel system that has shown superior inter- and intra-observer agreement to the Leighton-Rex scale, an early scoring system from 2011 (65–67). Standardization of reporting will support more accurate studies comparing bowel preparations to be undertaken.

## Patient preference for screening modalities

A screening test is considered effective not just when it has good sensitivity and specificity, but also when it is well accepted by the targeted population. The performance of colonoscopy as a screening tool for CRC is often hampered by low participation rates (68–70). Rates as low as 9% have been reported in certain subgroups within the Irish screening programme, Bowel Screen (69). Data from a recent pan-European screening report gave an overall 49.5% (range 22.8%-71.3%) uptake using FIT-based screening, falling significantly short of the recommended > 65% (70). The reason for such poor uptake has been investigated in numerous settings (71–73).

CCE does appear to be more acceptable than colonoscopy (21, 22, 27, 74–78). A recent interim analysis asked over 14,000 participants in a CRC screening programme (prior to their FIT result) whether they would prefer CCE or colonoscopy, with 50% choosing CCE versus 9% choosing colonoscopy (22). Of note, one recent systematic review with meta-analysis reported no statistical difference in patient preference, with 52% preferring CCE and 45% preferring colonoscopy (79). Tolerability was significantly higher for CCE and it was not clear to the authors why this did not translate into a preference for this modality. Disadvantages of CCE reported include longer wait time for results, unfamiliarity with the technology and the need for further procedures if pathology is detected (74, 79).

## Safety and cost

CCE is a safe procedure with the main considerations being capsule retention, and less frequently, capsule aspiration (44, 80–82). Adverse events reported, if any, are generally due to the preparation and include nausea, vomiting or bloating (21, 26, 33, 34, 67, 83, 84). These can occur in up to 25% (84). A 2017 meta-analysis of retention associated with capsule endoscopy found a retention rate of 2% in those referred for small bowel bleeding, and of 4-8% in those with suspected or known Crohn’s disease (81). In studies that performed a patency test prior to the capsule, retention rates were reduced by over 50% (85–87). Capsule aspiration is a rare but documented risk (82, 88, 89). A comprehensive review in 2017 estimated an overall aspiration rate of 0.1% (82).

An up-to-date review of the cost-effectiveness of CCE is needed. A 2010 cost-effective analysis of various CRC screening modalities concluded that PillCam COLON 2 was not a cost-effective alternative to FIT or colonoscopy, but advised their data was limited to the one study on the second generation capsule’s efficacy available at that time (90). A more recent study looked at the use of CCE in a population referred for CTC and found the cost-effectiveness of CCE to be favorable in this group (91). Recent ESGE guidance advised that CCE may be cost effective if it improves engagement in surveillance programmes (12). Hassan et al. similarly concluded if CCE improved initial compliance by 30% more than colonoscopy it would be a more effective and cost-effective approach (92).

## Challenges

CCE has been demonstrated to be comparable to traditional colonoscopy and better than CTC for detection of colonic pathology (19). It is safe and acceptable to patients. It is not without drawbacks, however, and certain areas, including bowel preparation and battery excretion rates as previously described, require particular attention.

Re-investigation rates remain high. Preliminary data from the Scottish Capsule Programme, (ScotCap) reported re-investigation rates of 63% in symptomatic patients and 70% in surveillance patients (76). The need for re-investigation is either the result of an incomplete examination or the need for polypectomy or biopsy by traditional sigmoidoscopy or colonoscopy. Further studies are needed to identify independent risk factors for incomplete CCE and to optimize patient pre-selection with a focus on low-risk groups (93).

Training in reading of capsules has not been formally incorporated into trainee programmes to date. Training standards are lacking and vary across different centers. One study showed the importance of skilled readers and how this influences outcomes, as expected (6). This lack of formal training has been previously noted, and more recently the ESGE published a position statement for small bowel capsule curriculum (94, 95).

The reading of colon capsules is, then, time consuming and currently a rate limiting step in the expansion of capsule services in hospitals. Vuik et al. reported a median time of 55 minutes for colon capsule reading (96). Although artificial intelligence software for colon capsules is currently in its infancy, it is anticipated that it’s use will allow faster reading of capsules, as well as improved PDRs (97). A recent systematic review of AI use in colon capsules recently included 9 studies (97). Though few, these studies show promising results for future integration of AI enhanced colon capsules into routine clinical practice (97–99).

## Discussion

Now the accuracy of CCE has been established, investment in training and artificial intelligence, along with continued robust data regarding efficacy are necessary to ensure CCE finds its place in routine clinical practice. Large scale initiatives, like the role out of colon capsule in the National Health Service (NHS) UK urgent cancer pathway and ScotCap, will provide a wealth of further information in the coming months and years (76). Outside of polyp detection and screening, there is also a growing body of evidence for the use of CCE in inflammatory bowel disease (IBD) which is to be further explored. ESGE recent 2020 guidance advises there is currently insufficient data to support use of CCE in diagnosis or surveillance of those with suspected or known IBD, however noted current preliminary data suggests it may be of use in monitoring of disease activity in UC (12).

Despite drawbacks, CCE is a viable diagnostic alternative to colonoscopy at an important time in service delivery. The increasing demand on colonoscopy waiting lists raises valid concerns regarding the ability of health services to deliver endoscopy in a timely fashion. It is clear that alternative diagnostic and surveillance modalities are necessary and that CCE has a central role to play (100).

## Author contributions

EG: Investigation, Writing – original draft, Writing – review & editing. OK: Writing – review & editing. BH: Writing – review & editing.
